# Money for Nothing? A Call for Empirical Evaluation of Biodiversity Conservation Investments

**DOI:** 10.1371/journal.pbio.0040105

**Published:** 2006-04-11

**Authors:** Paul J Ferraro, Subhrendu K Pattanayak

**Affiliations:** Zoological Society of LondonUnited Kingdom

## Abstract

The field of conservation policy must adopt state-of-the-art program evaluation methods to determine what works, and when, if we are to stem the global decline of biodiversity and improve the effectiveness of conservation investments.

For far too long, conservation scientists and practitioners have depended on intuition and anecdote to guide the design of conservation investments. If we want to ensure that our limited resources make a difference, we must accept that testing hypotheses about what policies protect biological diversity requires the same scientific rigor and state-of-the-art methods that we invest in testing ecological hypotheses. Our understanding of the ecological aspects of ecosystem conservation rests, in part, on well-designed empirical studies. In contrast, our understanding of the way in which policies can prevent species loss and ecosystem degradation rests primarily on case-study narratives from field initiatives that are not designed to answer the question “Does the intervention work better than no intervention at all?”

When it comes to evaluating the success of its interventions, the field of ecosystem protection and biodiversity conservation lags behind most other policy fields (e.g., poverty reduction, criminal rehabilitation, disease control; see Box 1). The immature state of conservation policy research is most clearly observed in the recent publication of the *Millennium Ecosystem Assessment*. While the biological chapters are rife with data and empirical studies, the *Policy Responses* volume [ 1] lists as one of its “Main Messages” the following: “Few well-designed empirical analyses assess even the most common biodiversity conservation measures.”

If *any* progress is to be made in stemming the global decline of biodiversity, the field of conservation policy must adopt state-of-the-art program evaluation methods to determine what works and when. We are not advocating that every conservation intervention be evaluated with the methods we describe below. We are merely advocating that *some* of the hundreds of biodiversity conservation initiatives initiated each year are evaluated with these methods. While there are challenges to field implementation of the methods, their use is no more expensive or complicated than biological assessments. Their promise lies in complementing case study narratives and testing intuition.

## Why Do We Need Evaluations?

Budgets for biodiversity conservation are thinly stretched [ 2], and thus judging the effectiveness of conservation interventions in different contexts is absolutely essential to ensuring that scarce funds go as far as possible in achieving conservation outcomes. Since the early 1990s, conservation projects have increasingly focused on “monitoring and evaluation.” This focus was stimulated by the desire of conservationists to be prudent in their use of scarce funds, and by the desire of donors, multilateral aid agencies, and international non-governmental organizations for greater transparency and accountability. In most efforts, overburdened and undertrained field staff tend to collect data on descriptive indicators (i.e., administrative metrics of change) instead of focusing on the fundamental evaluation question: what would have happened if there had been no intervention (a counterfactual event that is not observed)? Descriptive indicators can be important because they allow us to document the conservation process. However, we should be evaluating programs at a more fundamental level to find out whether, for example, conservation education workshops change behaviors that affect biodiversity. The focus must shift from “inputs” (e.g., investment dollars) and “outputs” (e.g., training) to “outcomes” produced directly because of conservation investments (e.g., species and habitats).

The field of program evaluation provides the tools to focus on outcomes [ 3–5]. Program evaluation uses randomized experimental policy trials and, when interventions are not randomly assigned, appropriate statistical tools to evaluate the effects of an intervention. Although the tools of program evaluation can achieve other objectives (e.g., help set priorities, adapt to new information), we wish to focus on the ability of these tools to measure *causes* of conservation outcomes because such a focus is absent in the conservation literature.

The field of conservation policy must adopt state-of-the-art program evaluation methods to determine what works and when. How many elephants would be poached if there had been no law banning ivory trade?

We are not the first to call attention to the need for evaluation of conservation interventions [ 6, 7]. Sutherland et al. [ 8] and Pullin and Knight [ 9] advocate an “evidence-based approach” that emphasizes meta-analysis as the main tool. Unfortunately, meta-analytic methods are premature in the field of biodiversity protection because there are few results to analyze in the literature. Others have noted the paucity of well-designed evaluations [ 10], called for learning from field projects [ 11], or reviewed trends and approaches in monitoring and evaluation approaches [ 12] (see http://www.conservationmeasures.org and http://fosonline.org). None, however, focus and elaborate on the key feature of the evaluation process whose absence is glaringly obvious and whose adoption would do the most good for distinguishing cause and effect in conservation initiatives: the measurement of *counterfactual* outcomes (see Box 2). We highlight the key elements of the state of the art in program evaluation and explain precisely why conservation science so desperately needs to adopt these methods.

## Status Quo: State-of-the-Practice in Conservation Science

Program evaluation is fundamentally a process of making inferences about an unobserved counterfactual event: what would have happened if there had been no intervention? For example, how much deforestation would we witness in a rainforest if there had been no conservation education in local villages? How many elephants would be poached if there had been no law banning ivory trade? Armed with a characterization of the counterfactual, a program evaluator can go beyond simple correlations to estimate the causal effect of interventions (be they projects, programs, or policies) on one or more outcomes.

Unfortunately, rigorous measurement of the counterfactual in the conservation literature is nonexistent. Consider some of the best-known conservation interventions—protected areas. Are such areas generally effective in protecting habitats and species? Based on observations that ecosystem conditions inside of protected areas are better than outside of protected areas [ 13] or management activities are positively correlated with perceptions of success by protected area managers [ 14], many conclude that protected areas are effective. However, such conclusions are premature without well-chosen counterfactuals that help us estimate what protected ecosystems would have looked like without protection. There is evidence that protected areas are often sited in areas that are not at risk for large-scale ecosystem perturbation [ 13, 15]. In other words, for political and economic reasons, protected areas are often located in areas with few profitable alternative uses of the ecosystem, and thus, even without protected status, the ecosystems would experience little degradation over time.

In their study of protected areas in Africa, Struhsaker et al. [ 16] write, “Contrary to expectations, protected area success was not directly correlated with employment benefits for the neighboring community, conservation education, conservation clubs, or with the presence and extent of integrated conservation and development programs.” Their results seem to question the effectiveness of the community-based interventions. However, interventions such as integrated conservation and development programs and conservation education are not randomly allocated across the landscape. Community-based interventions are more likely to be tried in areas that are experiencing high human pressures. Thus, comparing average conservation outcomes in areas where interventions benefit local people (high pressure) to average outcomes in areas where there are few such interventions (low pressure) gives a biased (down) estimate of the conservation effect of attempts to benefit residents around protected areas.

One of the “Main Findings” in the *Policy Responses* volume of the *Millennium Ecosystem Assessment* [ 1] is that “education and communication programs have both informed and changed preferences for biodiversity conservation and have improved implementation of biodiversity responses.” What is the evidence for this? Production of such evidence requires that the evaluation be built into the design of the original program and that data be collected on communities with and without education programs. We are not aware of a single case in which this type of evaluation has taken place. (Rare Pride campaigns have recently begun using control communities to evaluate the effects of their education campaigns, but have not yet analyzed and published the results [P. Vaughn, personal communication]).

When evaluating the effect of a conservation intervention, we must worry about confounding effects—effects that are contemporaneous with the intervention and could plausibly affect the outcome and thereby mask the intervention's effect. Examples of confounding effects include historical trends, unrelated programs or policies, and unobserved environmental and social characteristics. As in all scientific research, confounding effects are addressed through baselines, measures of covariates, and control groups [ 17]. Baselines measure pre-intervention conditions and behaviors, and thus control for initial conditions that may affect measures of program effectiveness. Covariates are observable factors that also influence the outcome measure; these factors may be socioeconomic, biophysical, economic, or institutional. Control groups are individuals, communities, or areas that do not experience the intervention but are otherwise similar (on average). Only by comparing sites or individuals with an intervention and those without can we make a convincing case for the intervention's effectiveness. Unfortunately, confounding effects have not been evaluated in much of the research on conservation interventions.

One potential confounder deserves mention because of its widespread, and apparently not well-understood, effects on our ability to make inferences about program effectiveness: endogenous selection. Current analyses typically do not consider the implications of why an area was picked for an intervention and another was rejected, or why some individuals “volunteered” and others did not. In any non-randomized program, characteristics that influence the outcome variable also often influence the probability of being selected into the program. Failure to address the issue of endogenous selection can lead to biased estimates of a program's effectiveness.

To better understand the problem of endogenous selection and the need for baselines, covariates, and controls, consider a currently popular conservation intervention: direct incentives in the form of Payments for Environmental Services (PES) [ 1, 18]. PES programs are being implemented globally in much the same way previous conservation interventions were implemented: with an unwavering faith in the connection between interventions and outcomes and without a plan to judge the effectiveness of such interventions. Say Costa Rica establishes a program to pay landowners who volunteer to maintain forest cover on their land. We might look at deforestation trends in Costa Rica before and after the program is implemented to evaluate the program's effectiveness. If deforestation rates were increasing before the program and are stable, declining, or increasing at a lower rate after the program is launched, we might be tempted to say the program is successful.

There are, however, two problems with this conclusion: it assumes that the past perfectly predicts the future and that “volunteers” represent the general population. If these assumptions are invalid, we cannot infer the deforestation rate in the absence of the program: the counterfactual is missing. With respect to the first assumption, there are good reasons to believe that past trends are not representative of future ones. Perhaps government subsidies that promote deforestation also declined around the same time that the payment program was initiated.

Comparing changes in forest cover among PES program participants *and* non-participants would avoid the assumption that the past perfectly predicts the future, but one still must ask, “Why did some landowners choose to participate and others did not?” For example, suppose one observes that forest cover on participating lands is much higher on average than that on non-participating lands. Can one conclude the program is effective? No. Participating landowners may be much more likely to have a pro-environmental ethic or low returns in alternative uses of the land. (For example, Langholz et al. [ 19] find that landowners with a pro-environment ethic are more likely to take advantage of Costa Rica's laws that allow for the establishment of private protected areas.) These same characteristics make the landowners less likely to deforest in the absence of the program. In a program that does not allocate payments randomly among interested landowners, we cannot simply compare the outcome of a participating landowner to that of the average non-participating landowner.

## State of the Art in Program Evaluation

How can researchers avoid the pitfalls described above and draw reliable inferences about causal effects? The evaluation literature emphasizes two alternatives for attributing effects to causes: experiments and quasi-experiments. Experiments identify the effect of an intervention by randomly distributing alternative causes over experimental conditions. Lacking this option, quasi-experiments carefully identify and study each plausible alternative cause and eliminate it through the design of the data collection or pattern matching in the data analysis [ 20]. We briefly review these experimental and quasi-experimental methods below. We can highlight only a few aspects of these methods because of space constraints, but we wish to emphasize that the methods are well developed and can be used *now* to evaluate conservation interventions. Without their widespread application, we will continue to be in the dark about the causal effects of our investments.

Scientists will appreciate the suggestion that one of the best approaches to reliable evaluations is to implement a field experiment in which an intervention is randomly assigned across individuals, communities, or regions [ 21]. If done correctly, this design ensures that potential confounders are balanced across intervention and control units and therefore any differences in the outcomes between the two can be attributed to the intervention. True random experimental designs are nonexistent in the field of conservation policy, but their absence has nothing to do with characteristics of the field. (There is, however, a long history of using randomized experimental designs to test biological hypotheses [ 22].) If economic development analysts can use field experiments to test the effects of micro-credit on household welfare and child deworming on school performance, there is no reason conservation practitioners cannot implement randomized experiments to examine the effectiveness of interventions such as payments for environmental services or conservation education.

The difference between what one can learn from a pilot initiative that uses an experimental (or quasi-experimental) design and from one that does not is enormous.

Although randomized experiments are possible, they can be challenging to implement and evaluate in many circumstances [ 23]. For example, it would be difficult to randomly regulate some communities and not others, and, for political reasons, it can be difficult to create a conservation program that provides benefits randomly rather than to areas that most need them. Thus, most conservation interventions will be implemented without randomization.

The lack of randomization does not imply that conservation scientists cannot rigorously evaluate and learn from conservation interventions. In thousands of evaluations in other disciplines, analysts use an array of quasi-experimental methods, grounded in theory and statistics, to isolate the causal effect of the intervention. Here we briefly describe three of the most popular quasi-experimental methods: (a) “natural” experiments, (b) instrumental variables, and (c) matching. Baker [ 24] provides a nontechnical general overview of these methods.

Closest in spirit to randomization is the method of natural experiments, which refer to situations where nature (or chance) creates “treatment” and “control” units [ 25]. In conservation biology, islands are the best known examples of natural experiments and have been important in testing ecological theories [ 26]. To test conservation policy effectiveness, one may, for example, have data from regions exposed to weather events such as hurricanes, floods, fires, or landslides, which create natural barriers that differentially protect or expose forests to social pressures. Areas on either side of these natural barriers provide comparable sites for evaluations. Nature, rather than people, selects the units on the basis of chance and therefore eliminates selection bias.

In the absence of random events, researchers can apply instrumental variable methods (IVM). Instrumental variables represent a source of *exogenous* variation in a conservation intervention. In the only published application of IVM in the conservation arena, Edmonds [ 27] considered how the devolution of forest management to local communities affects fuelwood extraction from local forests. He used the presence of extension programs and forest range posts as instruments to explain the *endogenous* formation of local management groups and found that communities with such groups extract less fuelwood from forests, on average. In general, good instrumental variables are difficult to find. Using IVM typically requires a mix of clear theoretical intuition, good quality secondary data, and a solid grasp of field conditions.

The method of matching is similar to IVM, but applies a different logic: areas (or landowners) that are in a conservation program are matched to otherwise “very similar” areas (or landowners) that are not in the program. These non-participating areas provide estimates of the counterfactual outcomes. Perhaps the best-known and most used matching method is propensity score matching [ 28]. Propensity scores represent the probability of participation in a conservation program, typically estimated from a statistical model of participation as a function of ecological, socio-economic, institutional, and geographic factors. Although there are no known published applications of matching-based evaluations of conservation outcomes, recent working papers use matching to evaluate the effects of forest disturbance on forest amenities, decentralized management on forest cover, and the Endangered Species Act on species recovery.

## If These Methods Are So Great, Why Isn't Anyone Using Them?

Given the billions of dollars invested in conservation initiatives and research in the past two decades, one may wonder why careful empirical studies and compelling data are lacking (see Box 3, however, for some recent examples). We do not claim to have conducted a formal study on this topic, but our experience in the field leads us to several conclusions.

First, one usually needs a remarkable combination of political will, a strong commitment to transparency, and a strong ethic of accountability to conduct a well-designed evaluation. Second, the diversity of donors and practitioners often leads to a plethora of objectives (e.g., scientific, aesthetic, humanitarian). Encouraging participants, including local actors, to agree on a set of explicit objectives to evaluate may be difficult in many conservation contexts.

At the very least, we must use the principles of evaluation to assess the potential for bias in making inferences about program effectiveness.

Third, conservation researchers are unaware of state-of-the-art empirical program evaluation techniques and the biases in current analyses. Donors and government agencies that fund conservation projects typically know little about program evaluation methods, and the practitioners who implement the projects typically lack incentives for careful analysis and falsification of hypotheses. Thus there is neither funding, nor a demand for funding, to conduct more careful analysis of interventions.

Fourth, many believe that rigorous evaluations of effectiveness are expensive and thus would divert scarce conservation funds toward “non-essential” investments. In contrast, researchers and practitioners in other policy fields have demonstrated that randomized experimental methods can be implemented in the context of small pilot programs or policies that are phased in over time. The difference between what one can learn from a pilot initiative that uses an experimental (or quasi-experimental) design and from one that does not is enormous.

Fifth, the nature of biodiversity conservation can make evaluations more difficult than in other fields. Where outcomes are local, strong and complex spillover effects can occur. Enforcement and cheating can be difficult to verify. Property rights are often unclear in low-income nations and so the effects of interventions are complex both cross-sectionally and in time-series. Biological outcomes often respond slowly to interventions (wildlife stocks), and only time-series identification can be used for many problems.

Sixth, many conservation interventions are short-term projects. The benefits of a careful evaluation, however, will largely be realized after the project ends and will accrue to the global conservation community. Field personnel are thus better off investing their time and resources in actions that will yield benefits to them rather than to the larger conservation community.

Seventh, program evaluation methods require data. In other fields of policy analysis, researchers have longstanding national surveys and historical relationships with government agencies and field practitioners that generate substantial datasets for research. Most conservation interventions, particularly in low-income nations, are framed as independent projects that “test” an idea in one or several locations. Data collection in these locations is often poor or non-existent, with little or no planning for data collection in control “non-project” locations. Furthermore, we can comprehensively link programs to changes in behaviors and conservation success only when we combine data on ecological, geographic, socio-economic, demographic, and institutional measures. Given the disciplinary biases about appropriate scale and methods for data collection, we rarely find such transdisciplinary efforts.

Finally, on a related point, credible estimates of conservation success depend on the ability to vary (or isolate) policy interventions in simple ways across space and time. We are well aware that within the same ecosystem, heterogeneity in institutions, income opportunities, access to markets, and other socio-economic characteristics can lead to different reactions to a given intervention. However, if every village or household is exposed to a different intervention (one gets direct payments, one gets fish farms, one gets agricultural assistance, etc.), we are left with few observations for each intervention and thus cannot make any inferences about effectiveness.

We are not proposing that all policy interventions be uniformly applied across space and time, but we are arguing that *some* policy interventions should be conducted in this manner to allow practitioners and decision makers to make inferences about their effectiveness. An evaluation may not be able to address the full range of questions, but addressing a tractable subset of questions may be far more productive, particularly given that reliable knowledge obtained from narrow studies may ultimately inform broader policy questions. Where it is impossible to use experiments, analysts must creatively use quasi-experimental methods to characterize the counterfactual and attribute cause to outcomes. At the very least, we must use the principles of evaluation to assess the potential for bias in making inferences about program effectiveness.

In the field of program evaluation, one lesson is paramount: you cannot overcome poor quality with greater quantity.

## Conclusions

What is the most effective way to slow deforestation? How can we reduce poaching of protected species in low-income nations? Does conservation education lead to changes in behaviors that affect biodiversity? We know little about the answers to these questions, but finding the answers is crucial to stemming the global decline of biodiversity and improving the effectiveness of conservation investments.

In the field of program evaluation, one lesson is paramount: you cannot overcome poor quality with greater quantity. We cannot learn from thousands of projects if none of these projects is designed in a way that permits an evaluation of its effectiveness. The results from a handful of well-designed individual tests can provide much more useful guidance than thousands of well-intentioned but poorly designed projects. In a field that takes the design and implementation of its initiatives seriously, we should expect to see greater value placed on evaluating the effectiveness of these initiatives.

Non-governmental organizations and governments can use the results of evaluations to focus their limited budgets on those programs that are most effective. Kremer [ 29] provides evidence that African non-governmental organizations in the education sector are not only embracing the need for evaluation but also serving as active partners in the design and implementation of quality evaluation, particularly by bringing in their local knowledge and grassroots mobilization capacity. With widespread uncertainty about the effectiveness of conservation investments, the provision of clear evidence on the effects of different interventions may also help spur support for more conservation financing.

Randomized policy experiments are often no more expensive than traditional “pilot” studies: the former simply builds program evaluation ideas into the project design. When randomization is not feasible and practitioners do not have the statistical skills to use appropriate quasi-experimental designs, we suggest a four-tiered rule for effectiveness evaluations (see Box 4). Each tier in this hierarchical system can make a vital contribution toward filling the large gap in our knowledge of what works.

As noted in the introduction, we are not advocating that every conservation intervention be evaluated with an experimental or quasi-experimental design, or that every project collect data on outcomes and covariates from treatment and control units before and after the intervention. We are merely advocating that some of the hundreds of millions of dollars that are invested each year in biodiversity conservation initiatives be spent in this manner. The fate of the world's ecosystems and species depends on it.

**Box 1. Example from the Development and Education Policy Literature**

Does reducing the cost of schooling increase student attendance? [ 30]

Initiated in the 1990s, the Mexican PROGRESA program provides cash grants to families if their children attend school regularly and receive preventative health care. The program was phased in randomly across villages. Analysts observed an average increase in enrollment of 3.4% for all students in grades 1 through 8, and 14.8% among girls who had completed grade 6. Using these same data, more sophisticated analyses were also done (“What would happen if the payments increased?”). In part, these clear and credible estimates of PROGRESA's effect led the Mexican government to expand the program, and other nations in Latin America to introduce similar programs.

**Box 2. Program Evaluation Terms**

**Counterfactual:** The outcome that would have happened if there had been no conservation intervention.

**Endogenous:** Used to describe a variable in a model or system that is causally dependent on other variables in the model or system.

**Exogenous:** Used to describe a variable in a model or system that is causally independent of other variables in the model or system.

**Selection bias:** Bias in estimating a program's effect that occurs when the participant and control groups differ from each other because of factors that also affect the program's outcomes. Such differences often arise when program units (species, acres, people, etc.) volunteer to participate in the program or are purposively inducted into the program. As a result, outcome differences between the participant and control groups may arise from differences between the groups rather than the program itself.

**Box 3. Examples of Ongoing Evaluations (Unpublished) of Conservation Initiatives**

Can cash incentives encourage upland farmers to forgo clear-cutting of forests?

A Vietnamese professor designed a quasi-experiment in which forest owners were offered cash to adopt sustainable forest management. Full randomization was considered difficult and costly. However, analysis of initial surveys showed that factors that affect forest use—e.g., availability of family labor, distance to roads—also affect decisions to participate, suggesting evidence of selection bias. Thus a control group of 50 households from a different upland community was chosen such that the characteristics of interest were balanced (i.e., no statistical difference) between “cash treatment” and the control groups. The control group received the forest management training, but not the cash payment. The ongoing experiment, while small and far from perfect, shows how an understanding of proper evaluation techniques can avert potential analytical pitfalls in the design stage (T. Bui Dung, unpublished data).

Does listing and funding under the U.S. Endangered Species Act affect species recovery?

The evidence marshaled to date for and against the effectiveness of the U.S. Endangered Species Act suffers from a problem common in analyses of biodiversity protection measures: the absence of data on what would have happened without the act. Statistical matching methods can be used to select control groups of species and thereby estimate how species listed and funded under the act would have fared had they not been listed or funded. The control groups must be similar in characteristics that can plausibly affect both listing/ funding and recovery (e.g., level of endangerment, biological characteristics, political influences, scientific knowledge, and advocacy). The analysis offers new insights and a methodology to guide evaluation of the effectiveness of non-randomized regulatory approaches to biodiversity protection (P. J. Ferraro, C. McIntosh, and M. Ospina, unpublished data).

Do protected areas improve health and income of local people?

Most answers to this question are based either on ex ante predictions from historical use patterns and strong assumptions, or ex post analyses that often prove only that the poor live near protected areas. Because national parks are not randomly sited, we can expect selection bias in interpreting the impact of parks on local people. An ongoing evaluation tracks health and livelihood outcomes of 1,000 households that traditionally have used resources around four new national parks in Gabon and 1,000 households that live outside the influence of the same parks. The simple selection of control households will go a long way to making a meaningful contribution in the debate over the effects of protected areas on local people [ 31].

**Box 4. A Four-Tiered Rule for Evaluating Conservation Interventions (Shown in Decreasing Priority)**

1. Consider ecological and socioeconomic factors that covary with the program.
2. Guess-estimate the direction of potential bias in interpreting intervention effectiveness.
3. Construct simple control groups (those that do not receive the intervention).
4. Collect data on outcomes and key inputs before and after interventions.

## Abbreviations

IVM: instrumental variable methods
PES: Payments for Environmental Services
